# The LIFESTYLE study: costs and effects of a structured lifestyle program in overweight and obese subfertile women to reduce the need for fertility treatment and improve reproductive outcome. A randomised controlled trial

**DOI:** 10.1186/1472-6874-10-22

**Published:** 2010-06-25

**Authors:** Meike AQ Mutsaerts, Henk Groen, Nancy CW ter Bogt, Johanna HT Bolster, Jolande A Land, Wanda JE Bemelmans, Walter KH Kuchenbecker, Peter GA Hompes, Nick S Macklon, Ronald P Stolk, Fulco van der Veen, Jacques WM Maas, Nicole F Klijn, Eugenie M Kaaijk, Gerrit JE Oosterhuis, Peter XJM Bouckaert, Jaap M Schierbeek, Yvonne M van Kasteren, Annemiek W Nap, Frank J Broekmans, Egbert A Brinkhuis, Carolien AM Koks, Jan M Burggraaff, Adrienne S Blankhart, Denise AM Perquin, Marie H Gerards, Robert JAB Mulder, Ed TCM Gondrie, Ben WJ Mol, Annemieke Hoek

**Affiliations:** 1 Department of Obstetrics and Gynaecology, University Medical Center Groningen, University of Groningen, Groningen, The Netherlands; 2 Department of Epidemiology, University Medical Center Groningen, University of Groningen, Groningen, The Netherlands; 3 Department of General Practice, University Medical Center Groningen, University of Groningen, Groningen, The Netherlands; 4 Centre for Prevention and Health Services Research, National Institute for Public Health and the Environment, Bilthoven, The Netherlands; 5 Department of Obstetrics and Gynaecology, Isala Clinics, Zwolle, The Netherlands; 6 Department of Obstetrics and Gynaecology, Free University Medical Center, Amsterdam, the Netherlands; 7 Department of Obstetrics and Gynaecology, Utrecht Medical Center University, University of Utrecht, The Netherlands; 8 Centre for Reproductive Medicine, Academic Medical Center, University of Amsterdam, Amsterdam, The Netherlands; 9 Department of Obstetrics and Gynaecology, Maastricht University Medical Center, University of Maastricht, Maastricht, The Netherlands; 10 Departement of Gynaecology and Reproductive Medicine, Leids University Medical Center, University of Leiden, Leiden, The Netherlands; 11 Department of Obstetrics and Gynaecology, Onze Lieve Vrouwe Gasthuis, Amsterdam, The Netherlands; 12 Department of Obstetrics and Gynaecology, Medical Spectrum Twente, Enschede, The Netherlands; 13 Department of Obstetrics and Gynaecology, Atrium Medical Center, Heerlen, The Netherlands; 14 Department of Obstetrics and Gynaecology, Deventer Hospital, Deventer, The Netherlands; 15 Department of Obstetrics and Gynaecology, Medical Center Alkmaar, Alkmaar, The Netherlands; 16 Department of Obstetrics and Gynaecology, Rijnstate Hospital, Arnhem, The Netherlands; 17 Department of Obstetrics and Gynaecology, Meander Medical Center, Amersfoort, The Netherlands; 18 Department of Obstetrics and Gynaecology, Maxima Medical Center, Veldhoven, The Netherlands; 19 Department of Obstetrics and Gynaecology, Leveste Hospital, Emmen, The Netherlands; 20 Department of Obstetrics and Gynaecology, Antonius Hospital, Nieuwegein, The Netherlands; 21 Department of Obstetrics and Gynaecology, Medical Center Leeuwarden, Leeuwarden, The Netherlands; 22 Department of Obstetrics and Gynaecology, Martini Hospital, Groningen, The Netherlands; 23 Department of Obstetrics and Gynaecology, Laurentius Hospital, Roermond, The Netherlands; 24 Department of Obstetrics and Gynaecology, Orbis Medical Center, Sittard, The Netherlands; 25 Department of Obstetrics and Gynaecology, Academic Medical Center, University of Amsterdam, Amsterdam, The Netherlands

## Abstract

**Background:**

In the Netherlands, 30% of subfertile women are overweight or obese, and at present there is no agreement on fertility care for them. Data from observational and small intervention studies suggest that reduction of weight will increase the chances of conception, decrease pregnancy complications and improve perinatal outcome, but this has not been confirmed in randomised controlled trials. This study will assess the cost and effects of a six-months structured lifestyle program aiming at weight reduction followed by conventional fertility care (intervention group) as compared to conventional fertility care only (control group) in overweight and obese subfertile women. We hypothesize that the intervention will decrease the need for fertility treatment, diminish overweight-related pregnancy complications, and will improve perinatal outcome.

**Methods/Design:**

Multicenter randomised controlled trial in subfertile women (age 18-39 year) with a body mass index between 29 and 40 kg/m^2^. Exclusion criteria are azoospermia, use of donor semen, severe endometriosis, premature ovarian failure, endocrinopathies or pre-existent hypertensive disorders.

In the intervention group the aim is a weight loss of at least 5% to10% in a six-month period, to be achieved by the combination of a diet, increase of physical activity and behavioural modification. After six months, in case no conception has been achieved, these patients will start fertility treatment according to the Dutch fertility guidelines. In the control group treatment will be started according to Dutch fertility guidelines, independently of the patient's weight.

**Outcome measures and analysis:**

The primary outcome measure is a healthy singleton born after at least 37 weeks of gestation after vaginal delivery. Secondary outcome parameters including pregnancy outcome and complications, percentage of women needing fertility treatment, clinical and ongoing pregnancy rates, body weight, quality of life and costs.

Data will be analysed according to the intention to treat principle, and cost-effectiveness analysis will be performed to compare the costs and health effects in the intervention and control group.

**Discussion:**

The trial will provide evidence for costs and effects of a lifestyle intervention aiming at weight reduction in overweight and obese subfertile women and will offer guidance to clinicians for the treatment of these patients.

**Trial registration:**

Dutch Trial Register NTR1530

## Background

There is indisputable evidence for the adverse effects of overweight and obesity on women's reproductive health. Overweight and obesity affect reproductive capacity in the general population [1] as well as in subfertile couples [2]. Ovulatory subfertile women with a body mass index (BMI) of 29 kg/m2 or higher have a 4% lower pregnancy rate per kg/m2 increase per year, compared to ovulatory subfertile women with a BMI below 29. In the Netherlands, approximately 30% of subfertile couples are overweight or obese. [3] Since childhood obesity is increasing, most notably among girls, a significant increase in obesity related subfertility can be anticipated in the future [4].

Overweight and obese women also have a lower live birth rate after IVF and ICSI [5-13], especially when these women are 36 years or younger [14]. A meta-analysis on the effect of overweight and obesity in artificial reproductive technologies (ART) reported a lower chance of pregnancy following IVF (OR 0.71, 95% CI:0.620-0.81) and an increased miscarriage rate (OR 1.3, 95% CI:1.06-1.68)[15].

Furthermore, pregnancies in obese women are associated with an increased risk of complications during pregnancy and delivery [16,17], causing an increase in maternal and neonatal morbidity and mortality [18,19]. There are more neonatal admissions [20] and five times higher costs [21].

In subfertile women lifestyle intervention could improve spontaneous conception chances and prevent unnecessary fertility treatment as well as obstetric complications. Observational and small intervention studies show that modest weight loss is associated with restoration of ovulation in anovulatory women and improves the likelihood of a pregnancy [22-24]. Weight loss can be achieved by lifestyle intervention programs incorporating the combination of a healthy diet, increase of physical activity and behavioural modification [25]. Weight loss has been advised for the improvement of reproductive function in overweight women, specifically with polycystic ovary syndrome (PCOS) [26,27]. In PCOS, insulin resistance and hyperinsulinism play a major role [28]. It has been shown that in women with PCOS even a modest weight loss improves this prediabetic state, and increases the rate of ovulation and the likelihood of a spontaneous achieved uncomplicated pregnancy [23,24,29,30]. However, the evidence of the effectiveness of weight reduction is still limited due to a lack of large controlled studies, and the effectiveness has not been established preceding ART.

At present, there are no evidence-based guidelines on fertility treatment in overweight and obese subfertile women. In the Netherlands, in some centers treatment is withheld in case of female overweight, and cut off levels for body mass index (BMI) differ among clinics. In other fertility centres overweight or obese women are treated irrespective of their BMI. The British Fertility Society advises to abstain from fertility treatment in women with a BMI over 35 kg/m^2 ^[31] and to start lifestyle intervention aiming on weight reduction, although there is not enough convincing evidence that weight reduction eventually leads to more spontaneous achieved uncomplicated pregnancies. Recently, a debate is started in literature whether or not restricting the access to fertility treatment on the ground of female body mass index. [32-35]

In view of the lack of convincing evidence from large intervention studies and the large practice variation in many countries, we designed a randomized controlled trial in overweight and obese subfertile women. In this trial, we will compare the costs and effects of a six-months structured lifestyle program followed by conventional fertility care as opposed to immediate conventional fertility care. We hypothesize that weight reduction improves spontaneous and treatment-related pregnancy chances, decreases overweight-related pregnancy complications and improves perinatal outcome.

## Methods

### Study design

This study is a multicenter randomised controlled trial in the Netherlands and inclusion started in July, 2009.

### Inclusion criteria

Subfertile women between 18 and 39 years who have a BMI between 29 kg/m^2 ^and 40 kg/m^2 ^are included. Subfertility is defined as failure to conceive within 12 months of unprotected intercourse in case of an ovulatory cycle, or in case of chronic anovulation due to WHO class I or II.

### Exclusion criteria

Couples suffering from azoospermia or using donor semen, women with endometriosis AFS class III or IV, chronic anovulation WHO class III (premature ovarian failure) or endocrinopathies (such as Cushing syndrome, adrenal hyperplasia and diabetes type I) will not be eligible for the study. Women with untreated pre-existent hypertension or with pregnancy induced hypertension, preeclampsia, eclampsia or HELLP syndrome in a previous pregnancy are also excluded. Patients who are unable to understand Dutch or to give informed consent will not be asked to participate in the study.

### Study management

All couples participating in the study will undergo a basic fertility work-up including a semen-analysis, monitoring of the cycle to assess ovulation and evaluation of tubal patency. After the work-up has been completed a prognosis for treatment independent pregnancy will be calculated using the Hunault model [36], followed by a management proposal for the individual couple.

Women eligible for the study will be referred to a research nurse for counselling and randomisation. The research nurse will not coach the patients during the lifestyle program.

Written informed consent is obtained before randomisation. Eligible women not willing to participate are registered as such.

### Randomisation

Randomisation is performed by a web-based randomisation program at a central randomisation center. Randomisation will be stratified according to participating center and ovulatory status.

### Intervention

#### 1a. The intervention arm

In the intervention arm, patients will participate in six-months during structured lifestyle program aiming at a weight loss of at least 5-10% of the original body weight. Nurses, dieticians or nurse practitioners who are trained prior to the study (intervention nurses) will guide and support them.

In the structured lifestyle program practice variation is minimized by using a structured software program, that has been evaluated previously [37].

As the combination of a dietary therapy, increased physical activity and an individualized behavioural modification plan leads to maximal weight loss and maintenance of weight loss[25,38,39] the lifestyle program targets at these three interventions:

### 1. Changing the dietary pattern

Women will be advised to adapt their dietary pattern and sustain a healthy diet with a caloric reduction of approximately 600kcal compared to their previous caloric intake (but not below 1200 kcal/day).

Self-monitoring is an essential tool to improve compliance during a lifestyle program. This will be implemented by using a web-based food diary http://www.voedingscentrum.nl, which gives feedback on food and caloric intake on a daily basis. Patients will be trained to use this device and the intervention nurse and the patient will together evaluate the daily caloric intake.

### 2. Stimulating physical activity of moderate intensity

Physical activity is necessary in order to obtain weight loss and increase the effect of dietary changes [25,38]. Physical exercise of moderate intensity (60-85% of maximum heart rate frequency) is advised during two to three times a week for at least 30 minutes. To increase physical activity in daily life, a pedometer (PAM; step counter) will be used aiming at 10.000 steps per day. To establish self-monitoring, a diary will be kept on these physical activities.

### 3. Changing behaviour

The motivation to change physical activity is monitored during the program by the PACE (Physician-based Assessment and Counseling for Exercise) score [40] which is part of the structured software program. This score measures the stages of change: precontemplation (not intending to change behaviour), contemplation (considering changing behaviour), preparation (making small changes in behaviour), action (actively engaging in behaviour change) and maintenance (sustaining the behaviour change over time). These stages are assessed at baseline, after 12 weeks and after 24 weeks of randomisation. Motivational counselling is individualized accordingly.

Changing behaviour is aimed for by motivational counselling which is directed at:

- Awareness of actual lifestyle leading to overweight or obesity.

- Counselling healthy lifestyle measures: the effect of healthy lifestyle in relation to subfertility and spontaneous and treatment dependent pregnancy chances, pregnancy complications and perinatal outcome.

- Formulating individualized goals embedded in a "patient contract". During the intervention individual goals will be evaluated, feedback will be given and goals will be adapted if necessary.

The lifestyle program consists of four sessions in the first three months and two additional sessions in the last three months. Four consultations by telephone or by e-mail are scheduled in between these sessions (Table 1)

**Table 1:** Time schedule lifestyle intervention


**Week**	**Consultation** **Outpatient, telephone or e-mail**	**Counseling subject lifestyle program**	**Duration (minutes)**

0	Randomisation	Baseline assessment^1,2 ^and explanation lifestyle program; handout of web based food diary, pedometer and activity diary. Hand out of (cost) questionnaires Blood sample	

1	Outpatient 1	Setting targets and planning goals; evaluating baseline measures^1,2^	45-60

3	Outpatient 2	Evaluating targets^1^	45-60

5	Telephone 1	Evaluating targets^1^	15

7	Outpatient 3	Evaluating targets^1^	30

9	Telephone or e-mail 2	Evaluating targets^1^	15

12	Outpatient 4	Evaluating targets^1,2^ Handout of (cost) questionnaires Blood sample	30

15	Telephone or e-mail 3	Evaluating targets^1^	15

18	Outpatient 5	Evaluating targets^1^	30

21	Telephone or e-mail 4	Evaluating targets^1^	15

24	Outpatient 6	Assessing targets and evaluation lifestyle program ^1,2^ Handout of (cost) questionnaires. Blood sample	30

> 24	Outpatient	Start subfertility treatment if applicable	

52		(Cost) questionnaires	

104		Cost questionnaire	


^1 ^Measuring weight, waist and hip circumference

^2 ^Measuring blood pressure

#### 1b. Start of fertility treatment in the intervention group

**Figure 1:**
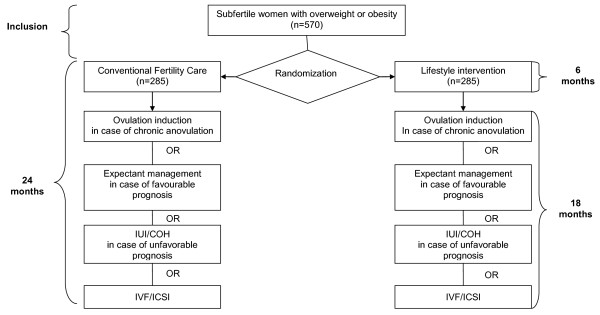
**Flowchart: Lifestyle study**.

As soon as patients in the intervention arm have finished their six-month lifestyle program, or have met their target weight reduction of 5-10% or when their BMI has decreased below 29 kg/m^2 ^, conventional fertility treatment will be started according to their individual prognosis based on the Hunault model [36]. If the estimated chance of spontaneous conception in ovulatory women is less than 30% in the forthcoming year, or when the couple has been subfertile for more than 3 years, fertility treatment is offered according to the guidelines of the Dutch Society of Obstetrics and Gynaecology (NVOG). Fertility treatment can either be IUI, IVF or ICSI, whatever is indicated according to the Dutch guidelines. When the Hunault model shows a prognosis of more than 30% pregnancy chance in the forthcoming year and patients are less than three years subfertile, expectant management will be proposed. In case of chronic anovulation, ovulation induction will be started using clomiphene or gonadotropins (Figure 1).

#### 2. The control arm

In the control arm, independently of the patient's weight, conventional fertility treatment will be started if the individual prognosis based on the Hunault model [36] is less than 30% chance of conception within the next year or when the couple has been subfertile for more than 3 years (guideline NVOG). Fertility treatment can either be IUI, IVF or ICSI, whatever is indicated according to the Dutch guidelines (NVOG). When the Hunault model shows a prognosis of more than 30% pregnancy chance in the forthcoming year and the duration of subfertility is less than three years, expectant management will be proposed. In chronic anovulatory patients, ovulation induction will be started using clomiphene or gonadotropins (Figure 1).

## Questionnaires and Follow-Up

All participating women (the intervention and the control arm) will complete several questionnaires: the SF-36 (measuring satisfaction) [41], the SQUASH list (for physical activity) [42] and the Dutch Eating Behaviour Questionnaire (DEBQ) for assessment of restrained, emotional and external eating behaviour. [43] In addition, questions will be asked on a women's food pattern (e.g. number and timing of meals), important food components (e.g. snacks, fruits, vegetables, sugar containing and alcoholic drinks and fatty acids content of food), portion sizes, and history of body weight (e.g. the duration of overweight and weight fluctuations). Finally, each participant will receive a questionnaire for details on associated direct costs of professional care and on indirect costs like travelling, sporting activity and productivity loss.

All mentioned questionnaires will be completed through a secured web based application. In case a patient decides not to use the web based application, she will receive paper questionnaires. The questionnaires will be completed at baseline, 12 weeks, 24 weeks and 52 weeks after randomisation and the questionnaire on costs also at the end of the follow-up period. Finally, patients who get pregnant during the period up to 24 months after randomisation will receive a questionnaire in which they can register their (self-reported) weight-gain during pregnancy. To assess gestational diabetes, an oral glucose tolerance test is advised for them at 28-30 weeks of gestation.

A structured case record form (CRF) is used to register reproductive outcome, fertility treatments as well as the course and outcome of subsequent pregnancies (including obstetrical interventions) for a period up to 24 months after randomisation.

### Withdrawal of individual patients

Patients can leave the study at any time for any reason if they wish to do so. Patients who drop out of the study will be asked to provide the reason for dropping out. This reason will be recorded and patients will be asked to provide information regarding the primary outcome (a healthy singleton born after at least 37 weeks of gestation after vaginal delivery) within 24 months after randomisation. In addition, they will be offered the possibility to continue completing cost questionnaires for the remainder of the study duration. Patients who drop out of the study will be treated according to the local protocols and guidelines for subfertility patients.

### Safety Monitoring Board

An independent Safety Monitoring Board (SMB), blinded for the study groups, will be installed to review complications related to fertility treatments, pregnancy, delivery and neonatal outcome. This board will evaluate reported complications after every 150 included patients and six months and twelve months after the end of the inclusion period (i.e. six times during the study). Furthermore, every case of preeclampsia, eclampsia or HELLP syndrome will be reported immediately to the SMB. The SMB will report its findings to the Medical Ethical Committee of the University Medical Center Groningen.

## Outcome Measures

### Main outcome measure

The primary endpoint will be a healthy singleton born after at least 37 weeks of gestation after vaginal delivery.

### Secondary study parameters/endpoints

Secondary outcome parameters are:

1. Pregnancy outcome and complications: miscarriage, multiple pregnancies, gestational diabetes, pregnancy induced hypertension, preeclampsia, HELLP syndrome, prematurity (< 37 weeks), macrosomia (birth weight > p90), induction of labour, prolonged duration of labour, surgical delivery (caesarean section), assisted delivery, peripartum increased blood loss (≥ 1000 ml).

2. Percentage of women needing fertility treatment in both groups (OI, IUI, IVF, ICSI) and clinical and ongoing pregnancy rates.

3. In case of IVF or ICSI: number and quality of oocytes, embryos and cryopreserved embryos.

4. Perinatal outcome: weight for gestational age, apgar scores, arterial pH, congenital anomalies, stillbirth, neonatal complications, and neonatal admission to a neonatal medium, high or intensive care unit.

5. Quality of life.

6. Additional parameters: weight changes, pre-pregnancy body weight, weight gain during pregnancy, waist circumference, behaviour influencing weight, i.e. nutritional habits and exercise pattern, blood pressure, glucose/insulin ratio (HOMA), hormonal profile (androgens, adipokines and cytokines).

7. Costs.

## Statistical Analysis

### Sample size

Based on the literature, the cumulative rate of healthy singletons born after at least 37 weeks of gestation after vaginal delivery during a follow-up period of two years is set at 45% for the control group[44]. We expect an improvement of healthy singletons born after at least 37 weeks of gestation after vaginal delivery from 45% to 60% in the intervention group. To demonstrate this difference of 15% between the two groups, 272 women (alpha 0.05, power 80%) are needed. To account for 5% loss to follow up and 20% drop out, in total 570 women (285 women per group) will be included.

To asses whether the groups are balanced, the study population will be compared for baseline measurements including female age, type of subfertility (primary or secondary), duration of subfertility, as well as sperm analysis according to WHO standards, subfertility diagnosis and initial BMI. Confounding factors, such as smoking and intoxications, will be addressed in the analysis.

### Univariate analysis and multivariate analysis

The primary analysis will be by intention to treat. The cumulative rate of healthy singletons born after at least 37 weeks of gestation after vaginal delivery in both groups will be compared using Kaplan-Meier analysis and the Log-rank test. In this analysis, patients will be censured at the time they discontinue or complete the study for other reasons than getting pregnant. In addition, pregnancy rates and 95% confidence intervals per group will be calculated based on the Kaplan-Meier estimates at various time points. Further analysis of delivery rates over time will be performed using Cox-regression analysis with correction for the stratification variables (i.e. ovulatory status and treatment centre) as well as for confounders. These analyses will also be performed for spontaneous and treatment-induced pregnancies separately. Exploratory subgroup analyses of the primary outcome will be performed for women with a BMI below 35 kg/m^2 ^versus above 35 kg/m^2 ^, as well as for anovulatory versus ovulatory women, for women with a waist-hip ratio of above 0.8 versus below or equal to 0.8 and for women who are 36 years or older versus younger than 36 years based on tests of statistical interaction with effect of treatment group.

The influence of HOMA, androgens, adipokines and cytokines on pregnancy chances and recovery of ovulations in anovulatory patients will be assessed using multivariate Cox-regression. Incidence of complications of treatment and during pregnancy will be compared in both groups using relative risks and 95% confidence intervals. Quality of life will be analysed using repeated measures analysis of variance. In the intervention group, we will analyse and identify the motivational factors at baseline which have a prognostic influence on the success of the lifestyle intervention.

A per protocol analysis will also be performed, in which patients who dropped out will be identified as non-compliant. Information on the primary outcome (i.e. a healthy singleton born after at least 37 weeks of gestation after vaginal delivery) within 24 months after randomisation, will be used whenever provided. Patients who drop out of the lifestyle intervention arm will be analysed in the conventional fertility care arm in this per protocol analysis.

### Economic evaluation

The aim of the economic evaluation is to compare the costs and health effects of the lifestyle program versus conventional fertility care, by a cost-effectiveness analysis with the proportion of healthy singletons born after at least 37 weeks of gestation after vaginal delivery as the primary outcome. These costs will be estimated in terms of costs per additional healthy singleton born after at least 37 weeks of gestation after vaginal delivery in a follow up period of 24 months.

The economic evaluation will be performed from a societal perspective. Direct medical and non-medical costs (intervention costs, time and travel costs) as well as indirect non-medical costs (productivity losses) will be taken into account.

The time horizon will be from randomisation to the end of follow-up. Resource use will be recorded as individual patient data in the CRF, with additional information from the cost questionnaires. Resource use will be valued according to Dutch guidelines. Intervention costs will be determined based on actual resource use obtained from the centers. Costs will reflect the resources of staff, materials, equipment, housing and overhead. Productivity loss will be valued by the friction cost method according to Dutch guidelines [45]. Costs of pregnancy and delivery will be calculated based on data from the literature [46].

Detailed information on maternal complications will be obtained from the patients medical files. Six weeks after the expected day of delivery all women will be contacted by telephone to obtain information about the delivery and the health of their child. If the child has been hospitalised, the responsible paediatrician will be contacted for further information.

Scenario analysis will be performed to model cost-effectiveness beyond the time horizon of the study. For longer term analyses, costs and effects will be discounted at commonly accepted rates. Sensitivity analysis will be performed on costs, pregnancy rates in the two groups and the valuation of different outcomes (no child, handicapped child, twin, healthy child, obstetric complications). Uncertainty surrounding cost-effectiveness estimates will be explored by bootstrapping.

## Ethical Considerations

The study is conducted according to the principles of the Declaration of Helsinki. The study protocol has been approved by the Medical Ethical Committee of the University Medical Centre Groningen.

The protocol is registered in the Dutch clinical trial register number NTR1530.

## Discussion

Specialists working in the field of reproductive medicine are frequently confronted with overweight and obese subfertile women. It is assumed that their pregnancy chances are reduced, and that they might benefit from weight reduction. In addition, weight reduction might lead to less pregnancy complications and consequently to better pregnancy outcome for mother and child. Therefore, BMI limits have been suggested for women undergoing fertility treatment, both with respect to patient's safety as well as treatment efficacy [15,31]. However, for most overweight and obese women weight reduction is hard to achieve and up till now, well powered studies addressing the issue of cost effectiveness of lifestyle intervention aiming at weight reduction in overweight and obese women with subfertility are lacking.

The present lifestyle study is the first large multicentre randomised controlled trial in which the costs and effects of a six-month during structured lifestyle program will be compared to conventional fertility care in overweight and obese subfertile women. The results of the study, which are expected in 2014, will help to make evidence-based guidelines for treatment in this patient group.

## Abbreviations

ART: Artificial Reproductive Technologies; BMI: Body Mass Index; COH: Controlled Ovarian Hyperstimulation; CRF: Case Record Form; DEBQ: Dutch Eating Behaviour Questionnaire; FSH: Follicle Stimulating Hormone; HELLP: Haemolysis, Elevated Liver Enzymes and Low Platelets; HOMA: Homeostasis Model Assessment; ICSI: Intra Cytoplasmic Sperm Injection; IUI: Intra-Uterine Insemination; IVF: In Vitro Fertilisation; NVOG: Nederlandse Vereniging voor Obstetrie en Gynaecologie; OI: Ovulation Induction; PACE: Physician-based Assessment and Counseling for Exercise; PAM: Physical Activity Meter; PCOS: Polycystic Ovary Syndrome; SF-36: Short Form 36; SMB: Safety Monitoring Board; SQUASH: Short Questionnaire to Asses Health; WHO: World Health Organisation.

## Competing interests

The authors declare that they have no competing interests.

## Authors' contributions

MM is responsible for the overall logistical aspects of the trial and drafted the manuscript. AH and BWM designed the trial and are responsible for the development of the protocol. AH applied for a grant and has overall responsibility for the trial. HG, NtB, JB, JL, WB, WK, PH, NM, RS and FvdV were involved in conception and design of the study. JL, WK, PH, NM, FdvV, JM, NK, EK, GO, PB, JS, YvK, AN, FB, EB, CK, JB, AB, DP, MG, RM, EG, BWM and AH are responsible for implementation of the study and inclusion of the eligible patients. All authors read and approved the final manuscript.

## Pre-publication history

The pre-publication history for this paper can be accessed here:

http://www.biomedcentral.com/1472-6874/10/22/prepub
